# Optimizing solar performance of CFTSe-based solar cells using MoSe_2_ as an innovative buffer layers

**DOI:** 10.1038/s41598-024-82309-7

**Published:** 2025-01-03

**Authors:** Mohamed Moustafa, Ziad Abu Waar, Shadi Yasin

**Affiliations:** 1 Department of Physics, School of Sciences and Engineering, The American University in Cairo, AUC Avenue, P.O. Box 74, New Cairo, 11835 Egypt; 2 Department of Physics, College of Science, The University of Jordan, Amman, 11942 Jordan; 3 College of Integrative Studies, Abdullah Al Salem University, Khaldiya, Kuwait

**Keywords:** SCAPS simulation, CFTSe, TMDCs, Buffer layer, MoSe_2_, Thin film solar cell, Solar energy, Solar cells

## Abstract

In this study, we explore the photovoltaic performance of an innovative high efficiency heterostructure utilizing the quaternary semiconductor Cu_2_FeSnSe_4_ (CFTSe). This material features a kesterite symmetrical structure and is distinguished by its non-toxic nature and abundant presence in the earth’s crust. Utilizing the SCAPS simulator, we explore various electrical specifications such as short circuit current (J_sc_), open circuit voltage (V_oc_), the fill factor (FF), and power conversion efficiency (PCE) were explored at a large range of thicknesses, and the acceptor carrier concentration doping (N_A_). Our results demonstrate that optimized parameters yield a remarkable PCE of 26.47%, accompanied by a V_oc_ of 1.194 V, J_sc_ of 35.37 mA/cm^2^, and FF of 62.65% at a CFTSe absorber thickness of 0.5 μm. Furthermore, the performance of the photovoltaic cell is assessed for the defect levels in the CFTSe absorber and MoSe_2_ buffer layers. Results indicate that deep defect levels above 1 × 10^17^ cm^− 3^ lead to a decrease in J_sc_. The study also investigates the effect of operating temperature on cell performance within the 300–500 K range. A notable decline in V_oc_ is observed, likely due to an increase in saturation current, suggesting an interaction between temperature and cell behavior. In this work, we propose a practical CFTSe-based structure that replaces conventional buffer layers, such as CdS, with MoSe_2_ TMDC as a promising alternative buffer layer, paving the way for more sustainable solar technology.

## Introduction

As the global population continues to grow, energy demand rises correspondingly. With limited energy sources capable of meeting the projected terawatt-scale demand by 2050, solar energy stands out as a vital alternative. It has the potential to offer a solution to the global energy crisis while addressing environmental concerns. One of the most effective ways to harness solar energy is through solar cells, which convert sunlight directly into electricity^1^. Current research focuses on developing solar cells that combine cost-effectiveness with high photoelectric conversion efficiency. This includes silicon, Perovskite and double perovskite halide-based solar cells^2–4^, thin film Cadmium Telluride (CdTe), Copper Indium Diselenide (CIS), and Copper Indium Gallium Diselenide (CIGS), which have reached record efficiencies of up to 21.7% and 21.5%, respectively^5–9^. Recently,  Gohri et al.^10^ achieved an impressive efficiency of 25.2% by employing multiple grading profile analysis with a CIGS thickness of 1 μm. However, these materials face significant challenges due to the toxicity of cadmium and selenium and the low availability of tellurium and indium^11,12^. To address these issues, alternative materials, specifically quaternary semiconductors like Cu_2_ZnSnS_4_ (CZTS), Cu_2_ZnSnSe_4_ (CZTSe), Cu_2_FeSnS_4_ (CFTS), and Cu_2_FeSnSe_4_ (CFTSe), have gained widespread attention for several reasons including non-toxicity, an abundance of earth-friendly components, and suitable band gap (1.4–1.6 eV)^13–17^.

CZTS, CZTSe, and CZTSSe have been extensively studied for their cost-effectiveness and efficiency in thin-film solar cells. For instance, a champion CZTSe solar cell achieved an impressive power conversion efficiency of 12.6% using a spin-coating process and CdS as a buffer layer^18^. Recently, a silver-doped graded CAZTS solar cell incorporating Sn_2_S_3_ as a BSF layer has been investigated achieving 27.3% efficiency^19^ Additionally, reference^20^ provides a comprehensive review of kesterite-type CZTS thin-film solar cells, highlighting their current trends and developments. Conversely, CFTSe has not been as widely explored, possibly due to the relatively limited research and development efforts focused on CFTSe compared to other materials. Despite this, CFTSe holds potential for future solar cell technologies. For instance, Zhou et al. detailed the synthesis of CFTSe microparticles as a photovoltaic absorption material, utilizing an atmospheric pressure liquid reflux method^21^. Further research and development could enhance the efficiency and stability of CFTSe-based solar cells, potentially unlocking their full capabilities as a viable alternative to other thin-film photovoltaic materials. CFTSe exhibits a high absorption coefficient of 10^5^ cm^−1^ and a suitable direct bandgap from 1.1 to 1.4 eV, aligning well with the solar spectrum^22–24^. The elements Fe and Sn in CFTSe are earth-abundant, making it a promising candidate for photovoltaic applications. Comprising abundant, low-cost, and non-toxic elements such as copper, zinc, tin, and selenium, CFTSe is environmentally friendly and suitable for the mass production of solar cell devices, unlike other thin-film solar cells. CFTSe is an environmentally safe and non-toxic material. It is a p-type semiconductor and possesses a stable crystal structure, which allows the film to produce a good performance. The stannite structure of CFTSe further enhances its performance. Additionally, its stability improves with increasing temperature, making it a robust material for solar applications^22–24^.

While CFTSe shows great promise as a high-performance absorber material for thin-film solar cells, optimizing the buffer layer remains a challenge for further enhancing cell performance. The buffer layer, typically an n-type material, plays a crucial role in forming the p-n junction in photovoltaic devices. Traditionally, CdS has been a common choice for this layer, significantly improving solar cell efficiency^25^. However, cadmium is toxic and restricted in electronics, and CdS has a bandgap of approximately 2.40–2.50 eV, which limits optical absorption in the short wavelength range and overall solar cell performance. In response to these challenges, significant efforts have been directed toward finding alternative, non-toxic buffer materials. Potential candidates include ZnS, Ins, ZnSe, Zn(O, OH), Zn(O, S, OH), and ZrO^26–28^. The use of transition metal dichalcogenides (TMDCs), such as MoS_2_, as buffer layers for CZTS and CIGS solar cells has been explored^28,29^. Building on this trend, we propose MoSe_2_ as a novel and potentially superior buffer layer for CFTSe solar cells.

Molybdenum diselenide (MoSe_2_) is a group VI transition metal dichalcogenide (TMDC) known for its chemical inertness and a suitable bandgap ranging from 1.0 eV to 1.5 eV, coupled with high hole mobility. It has been extensively studied for its potential benefits in various applications, especially as a thin film. The electronic band structure of MoSe_2_, along with other TMDCs, has been studied both experimentally and through calculations using diverse methods^30–33^. TMDCs, characterized by their MX_2_ structure—where metal atoms are sandwiched between layers of chalcogens—exhibit a range of electronic properties from metals to insulators. They possess unique morphology with thin, flexible, high-quality surfaces free of dangling bonds, making them ideal for thin-film applications. Their strong in-plane covalent bonding and weak Van der Waals forces between layers contribute to their stability^34^. Using TMDCs as buffer layers helps resolve problems like lattice parameter mismatch and differences in thermal expansion coefficients between the absorber and buffer layers, which can significantly affect cell performance. Recently, TMDCs, including the MoSe_2_, have garnered significant attention due to their low cost and exceptional electrochemical, optical, mechanical, and thermal properties, as well as their environmental friendliness. These attributes position them as promising candidates for enhancing solar cell efficiency^35–40^.

This paper utilizes SCAPS software to investigate a novel thin-film solar cell (CFTSe) structure featuring a MoSe_2_ buffer layer. It examines the impact of incorporating MoSe_2_ TMDC as a new buffer layer on solar cell performance. The presence of defects significantly influences the optical and electrical parameters of semiconductor materials, highlighting the importance of defect analysis in enhancing the output characteristics of photovoltaic cells. After optimizing cell thickness and considering defects within each active absorber layer, the paper discusses the resulting photovoltaic parameters. Additionally, it explores the effect of high ambient temperature conditions on the overall performance of CFTSe. Practical results indicate variations in all electrical parameters of the solar cells with changing temperatures.

**Fig. 1:**
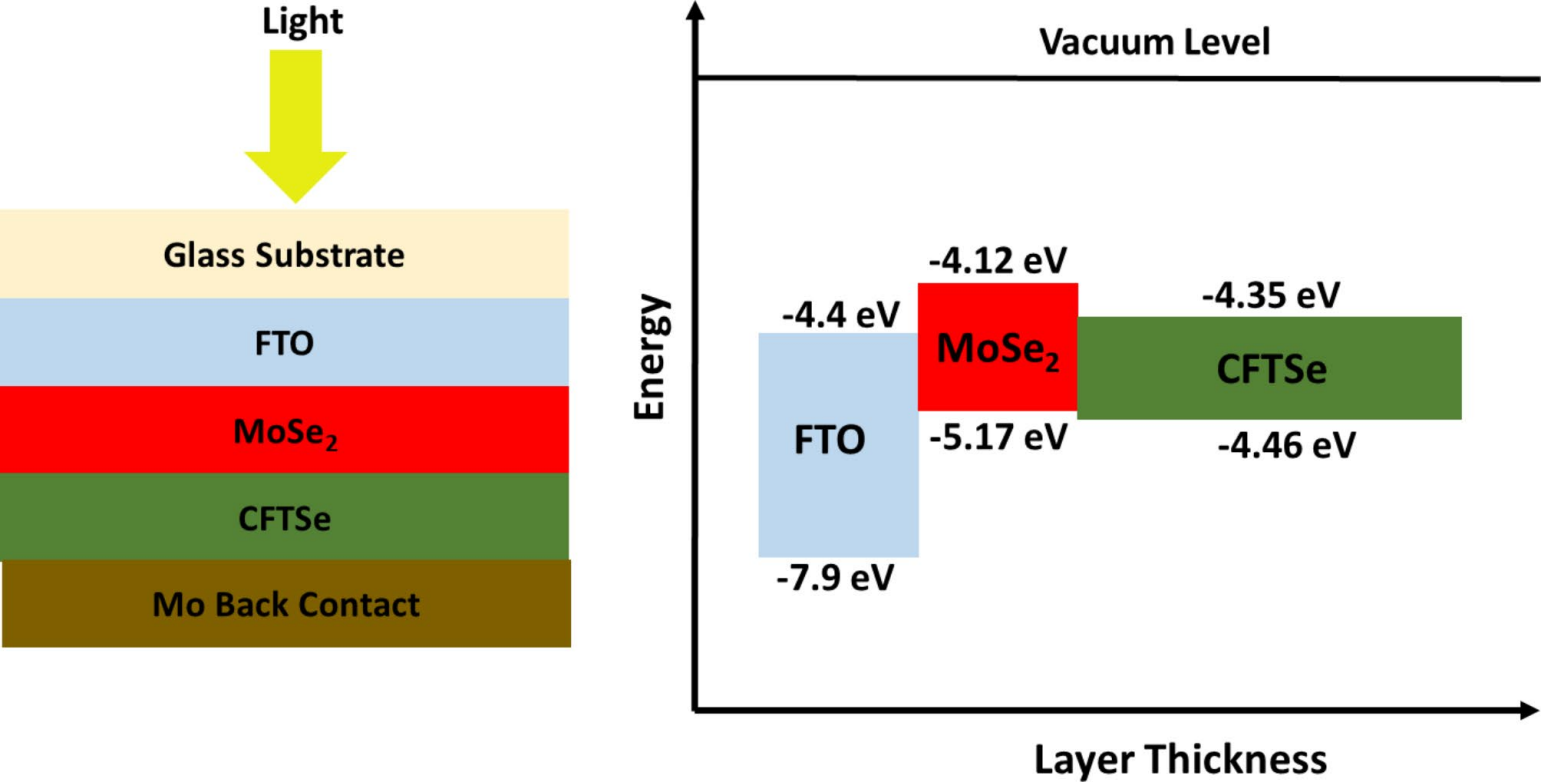
The solar cell structure of the modeled CFTSe-based solar cell with MoSe_2_ Buffer Layer and its corresponding energy band diagram.

## Simulation techniques and material properties

This study investigates the photovoltaic performance of Cu_2_FeSnSe_4_ (CFTSe) using the SCAPS-1D solar cell simulation software^39–41^. SCAPS is a powerful and versatile simulation tool known for its effectiveness across various solar cell technologies, including Perovskite, CIGS(Se), CZTS(Se), CdTe, organic, and polymeric cells^41–47^. It can model both heterojunction and multi-junction photovoltaic devices, delivering results that align closely with experimental data, thus establishing it as a reliable choice for researchers. SCAPS enables simulations of solar cell structures with up to seven layers, accommodating variations in layer thickness, energy bandgap, electron affinity, defect density, and doping levels under different AC and DC testing conditions. For this study, simulations are performed under standard testing conditions: AM 1.5 solar spectrum, 1000 W/m^2^ light intensity, and an operating temperature of 298 K. Figure 1 depicts the schematic configuration of the CFTSe solar cell used in this study. The cell structure consists of three layers: FTO Window Layer: This layer has a relatively large energy bandgap to permit light transmission to the subsequent layers. MoSe_2_ Buffer Layer:

The MoSe_2_ layer, with its medium bandgap and electron affinity, mitigates surface defects between the window and absorption layers, improving photon access. It does this by passivating defects, such as selenium vacancies, and chemically interacting with surface defects on the CFTSe layer. Its smooth, layered structure forms a clean, well-ordered interface with CFTSe, reducing interface trap states and enhancing heterojunction performance. Additionally, the MoSe_2_ layer creates a uniform interface, minimizing surface roughness and defects, which improves the overall junction quality and device performance. Further details on defect effects are discussed later in the manuscript. Featuring a medium energy bandgap and suitable electron affinity, the MoSe_2_ layer mitigates surface defects, enhancing photon absorption. This occurs through several mechanisms, including defect passivation, where MoSe_2_ chemically interacts with surface defects like selenium vacancies or dangling bonds on the CFTSe layer^48^. Its quasi 2-D layered structure and smooth surface enable MoSe_2_ to form a well-ordered, clean interface with CFTSe, reducing recombination centers and boosting heterojunction performance^48^. Additionally, the MoSe_2_ layer smooths the interface with CFTSe, minimizing surface roughness and defects, thus improving junction quality and overall device efficiency. Further details on the effects of defects on solar cell performance will be elaborated later in this manuscript. The typical CFTSe-based solar cell structure includes a p-type wide-bandgap absorber layer deposited on a molybdenum (Mo) coated back glass substrate, accompanied by an n-type buffer layer and a window layer. MoSe_2_, a layered semiconductor, can exhibit either n-type or p-type conductivity with a net carrier concentration of approximately and Hall mobilities of about 154 cm^2^/Vs at 298 K. The material properties used in the current simulation are summarized in Table 1^42,49–51^.

**Table 1:** Input parameters used for simulation^42,50,51^.

Parameter	Symbol (unit)	FTO	MoSe_2_	CFTSe
Layer thickness	d (nm)	50	50–200	500–2500
Bandgap	E_g_ (eV)	3.5	1.20	1.11
Electron affinity	χ (eV)	4	4.12	4.35
Dielectric permittivity	K	9	8.76	9
Effective density of states	N_c_ (cm^-3^)	1 × 10^19^	2.8 × 10^19^	2.2 × 10^18^
Effective density of states	N_v_ (cm^-3^)	1 × 10^18^	2.65 × 10^19^	1.8 × 10^19^
Electron thermal velocity	(cm/s)	1.0 × 10^7^	1.0 × 10^7^	1.0 × 10^7^
Hole thermal velocity	(cm/s)	1.0 × 10^7^	1.0 × 10^7^	1.0 × 10^7^
Electron mobility	(cm^2^/V.s)	20	100	4.58
Hole mobility	(cm^2^/V.s)	10	25	4.58
Shallow uniform donor density	N_D(sh)_ (cm^-3^)	1 × 10^18^	1 × 10^18^	0
Shallow uniform acceptor density	N_A(sh)_ (cm^-3^)	0	0	8.01 × 10^20^
Defect density	(cm^-3^)	1 × 10^14^	1 × 10^14^	1 × 10^14^

## Results overview and discussion

**Fig. 2:**
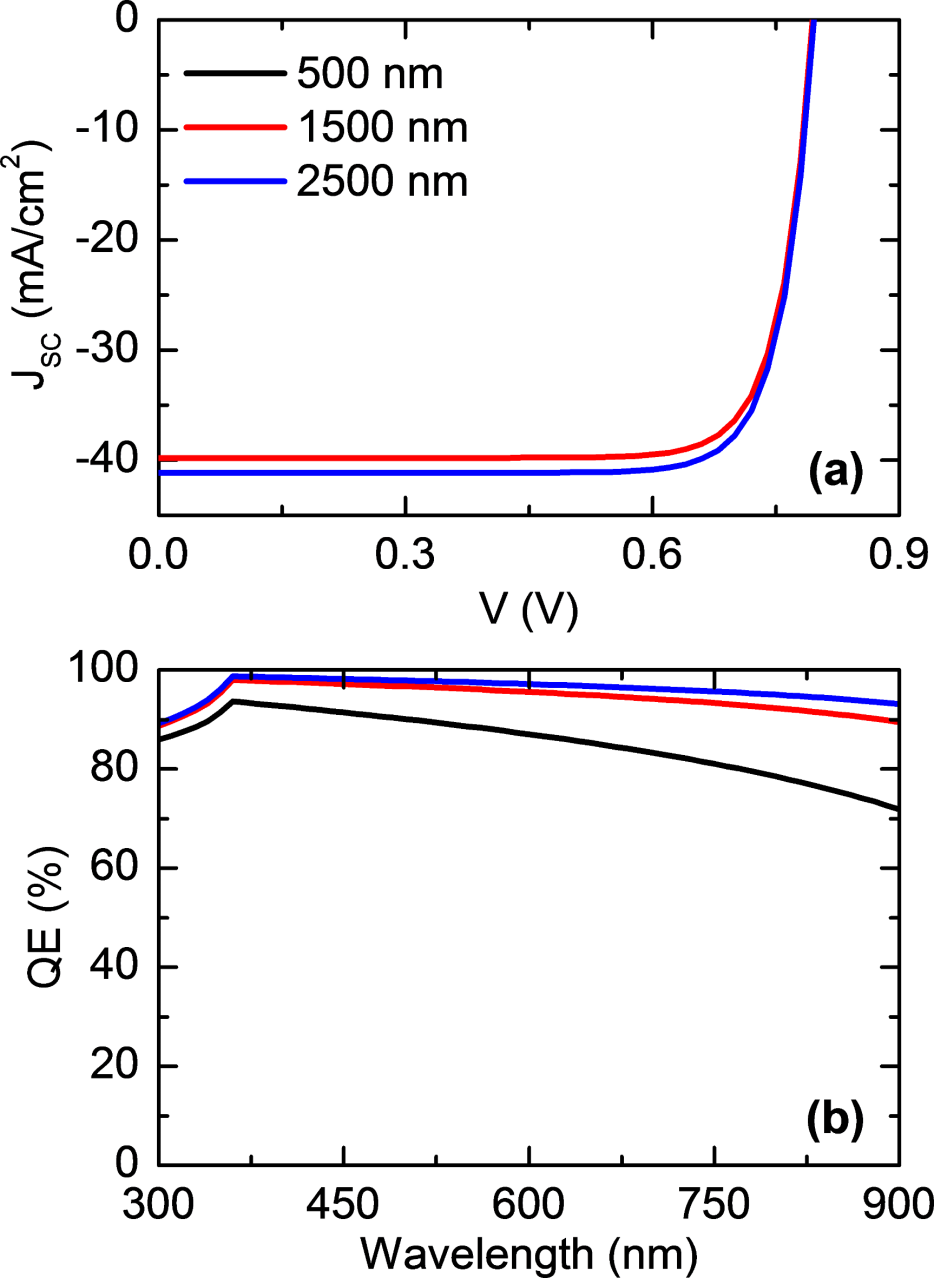
The obtained (**a**) Current–Voltage (J–V) and (**b**) quantum efficiency (QE) characteristics of the CFTSe-based solar cell at three different absorber layer thicknesses.

Figure 2 (a) shows the current-voltage (J–V) characteristics of the solar cell for three different thicknesses of the CFTSe-based absorber layer: 500 nm, 1500 nm, and 2500 nm. For the 2500 nm thickness, the initial simulation parameters are a J_sc_ of 34.09 mA/cm^2^, a V_oc_ of 0.789 V, an FF of 80.98%, and a PCE of 21.78%. Figure 2 (b) shows the quantum efficiency (QE) as a function of wavelength. QE, defined as the ratio of the photogenerated current I (λ) to the incident photon flux φ_p_(λ), where q is the elementary charge, shows a significant increase at short wavelengths (300 nm to 600 nm) for the optimized cell. In contrast, QE decreases at longer wavelengths beyond 700 nm, with a more pronounced drop for the 500 nm absorber thickness. This reduction is likely due to incomplete absorption of long-wavelength photons. From Fig. 2 (a), it is clear that increasing the absorber layer thickness enhances the solar cell’s performance, as indicated by the steeper J-V curve with a greater slope (∆I/∆V). These results highlight the importance of absorber thickness in reducing series resistance and improving overall efficiency.

To enhance the discussion on device output performance, particularly the J–V curve and achieved efficiency, it’s essential to calculate the lattice mismatch between the absorber and buffer layers using the following equation^52^:

1 $$\:\delta\:=2\left|{a}_{s}-{a}_{e}\right|/{a}_{s}-{a}_{e}$$

where δ is the lattice mismatch, $\:{a}_{s}$ and $\:{a}_{e}\:$are the substrate and epitaxial thin film lattice constants, respectively. The values of the lattice constants of the different materials utilized in this analysis have been sourced from prior research^53–56^. Understanding the lattice mismatch is critical in solar cells, as it can influence the overall performance of the solar cells. A large lattice mismatch can compromise interface stability, leading to poor charge carrier collection and reduced efficiency. Table 2 summarizes the calculated $\:\delta\:$ between CFTSe and different buffer layers, including MoSe_2_. As shown in Table 2, the lattice mismatch between CFTSe and MoSe_2_ is 9.22%, which is lower than that of the other proposed buffer layers listed. Indeed, TMDCs can accommodate differences in lattice parameters due to their unique layered structure and weak vdW forces between the layers. This allows for better alignment at the interface, minimizing strain and reducing defect density. The reduced lattice mismatch between CFTSe and MoSe_2_ contributes to a decrease in interface defect density and recombination losses, ultimately leading to enhanced performance parameters. Incorporating MoSe_2_ as a buffer layer thus plays a crucial role in improving the overall efficiency and effectiveness of the solar cell device.

**Table 2:** Lattice mismatch at CFTSe/buffer interface with different layers compared to MoSe_2_.

Layer	Lattice parameters			Lattice mismatch
	*a* (Å)	*b* (Å)	*c* (Å)	
CFTSe	5.69	5.69	11.32	–
MoSe_2_	3.32	–	12.29	9.22%
ZnSe	4.00	4.00	6.57	19.38%
CdS	4.135	–	6.749	24.65%

**Fig. 3:**
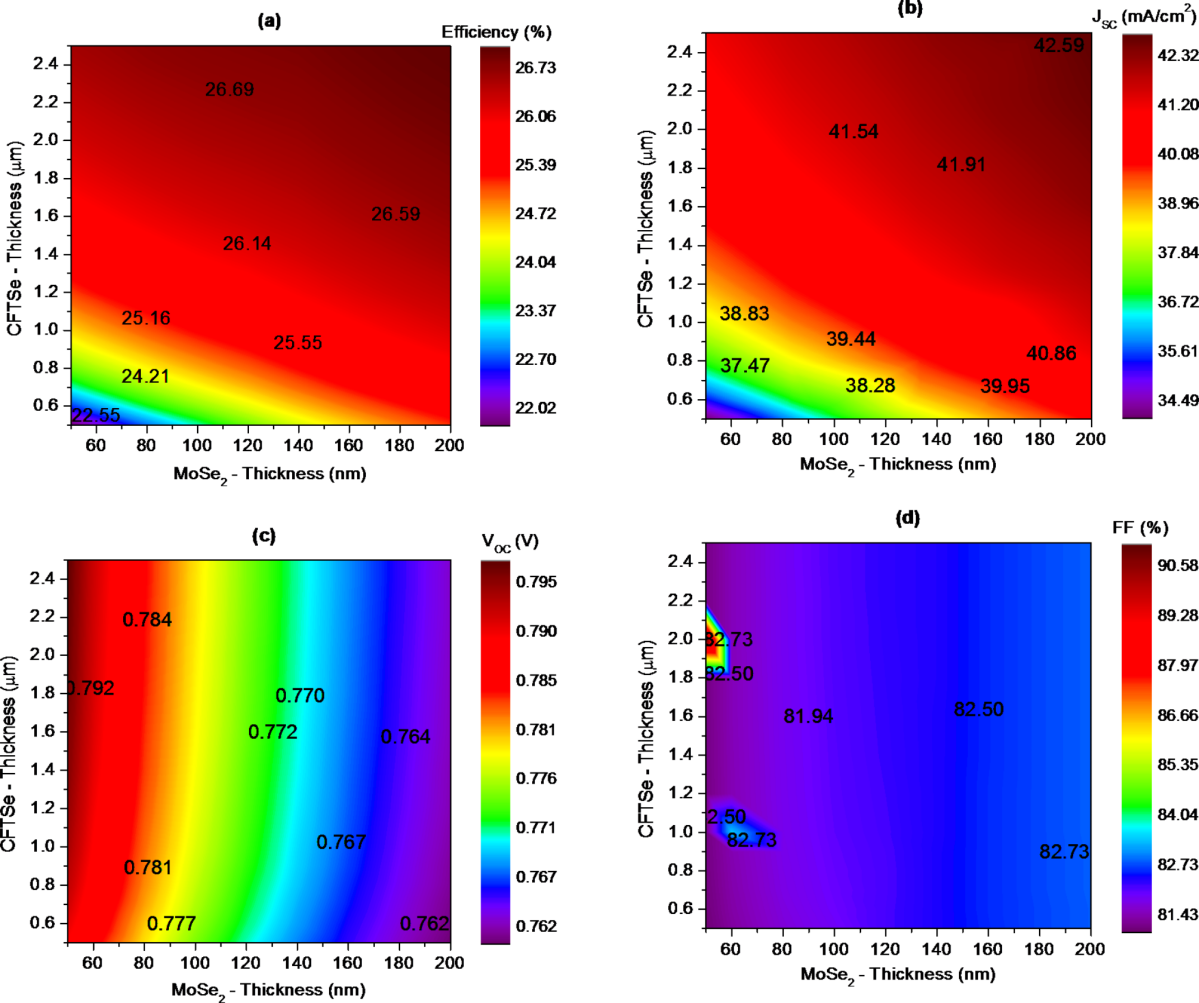
Concurrent effect of MoSe_2_ buffer layer (*x*-axis) and CFTSe absorber (y-axis) thicknesses on the photovoltaic performance of CFTSe-based solar cells.

A comprehensive analysis was conducted to examine how varying the absorber thickness and incorporating MoSe_2_ as a novel buffer layer influence the performance of solar cells. The thickness of the Cu_2_FeSnSe_4_ (CFTSe) absorber layer is pivotal for optimizing solar cell efficiency. It must be meticulously optimized to enhance photon absorption while avoiding excessive thickness that could compromise the control over reverse saturation current. The study investigated varying the thickness of the CFTSe absorber layer from 500 nm to 2500 nm and the MoSe_2_ buffer layer from 50 nm to 200 nm. Figure 3 presents the photovoltaic parameters, including open-circuit voltage (V_oc_), short-circuit current density (J_sc_), fill factor (FF), and conversion efficiency (PCE). Optimal performance was achieved with a MoSe_2_ buffer layer thickness of 200 nm and a CFTSe absorber layer thickness of 2500 nm. Under these conditions, the solar cell exhibited a notable efficiency of 27%, a J_sc_ of 42.75 mA/cm^2^, a V_oc_ of 0.763 V, and an FF of 82.80%. The data reveal significant performance improvements with increased absorber layer thickness. For instance, increasing the CFTSe thickness from 500 nm to 2000 nm, while keeping the MoSe2 layer at 50 nm, resulted in an increase in Jsc from 34.09 mA/cm^2^ to 41.14 mA/cm^2^, with the FF remaining stable at 81.1% and PCE rising from 21.78 to 26.58%. Voc showed a slight increase from 0.789 to 0.797 V. Beyond a CFTSe thickness of 2000 nm, there were no substantial gains in performance, indicating that a thickness of 2000 nm is sufficient for achieving optimal efficiency. Further increases in thickness would only lead to excessive material use without additional performance benefits. The performance trends observed with varying absorber thickness can be largely attributed to the effects of back contact recombination. Photogenerated carriers are prone to recombination at the back contact, which reduces their contribution to quantum efficiency. Increasing the absorber layer thickness allows for the generation of more photogenerated carriers, which can be collected before recombination, thereby improving efficiency. Thicker absorber layers also decrease the back contact recombination current density by placing the back contact further from the depletion region. However, it is important to avoid excessive increases in absorber thickness. While a thickness of up to 2.0 μm improves efficiency, further increases lead to diminishing returns. Beyond this thickness, incoming photons may be absorbed deep within the absorber layer, far from the depletion region, resulting in carriers that may not reach the space charge region within their lifetime^57^. This underscores the need for optimization to minimize bulk recombination. Excessively thick absorber layers, while reducing back contact recombination, can lead to reduced efficiency due to increased bulk recombination effects^58,59^.

Analyzing the impact of the MoSe_2_ buffer layer thickness on photovoltaic cell parameters reveals a notable improvement in J_sc_ and overall cell efficiency as the buffer thickness increases. Specifically, J_sc_ increases from 34.09 mA/cm^2^ to 39.90 mA/cm^2^, when the MoSe_2_ buffer layer thickness increases from 50 nm to 200 nm, while maintaining a constant absorber thickness of 0.5 μm. This enhancement in Jsc can be attributed to the increased number of ionized atoms in the thicker buffer layer, which facilitates more efficient charge carrier collection. This improvement in charge carrier collection contributes significantly to the overall enhancement of cell performance, demonstrating the importance of optimizing buffer layer thickness for achieving higher efficiency in photovoltaic cells. Conversely, V_oc_ slightly decreases as the MoSe_2_ buffer layer thickness increases. For instance, V_oc_ drops from 0.789 V to 0.761 V when the buffer layer thickness increases from 50 nm to 200 nm, while keeping the absorber thickness fixed at 0.5 μm. This reduction in V_oc_ is attributed to photon losses in the thicker buffer layer at moderate doping concentrations. As the n-type MoSe_2_ buffer layer becomes thicker, more incident photons are absorbed by the buffer layer itself, resulting in fewer photons reaching the absorber layer. This leads to a reduction in the generation of electron-hole pairs and, consequently, a decrease in V_oc_. However, both PCE and FF improve with increasing buffer layer thickness. For example, PCE rises from 21.78 to 25.12% at the same buffer thickness range. The simulated optimal values are achieved by an FF of 82.78% and a PCE of 25.12%.

The results can be interpreted as follows: In heterojunction solar cells, the buffer layer forms a junction with the absorber layer, facilitating the maximum penetration of incoming light into the absorber layer. A thicker buffer layer increases photon absorption within the buffer itself, reducing the number of photons that reach the absorber layer and causing higher photon loss. This reduction in photon flux to the absorber layer results in decreased quantum efficiency of the solar cell^60^. An excessively thick buffer layer can also lower carrier separation rates^61^. Conversely, a buffer layer that is too thin may allow forward leakage current to the front contact, adversely affecting cell performance^62^. For optimal experimental performance, it is recommended to use a MoSe_2_ buffer layer thickness in the range of 150 nm to balance performance and minimize the issues associated with both excessively thick and thin buffer layers.

**Fig. 4:**
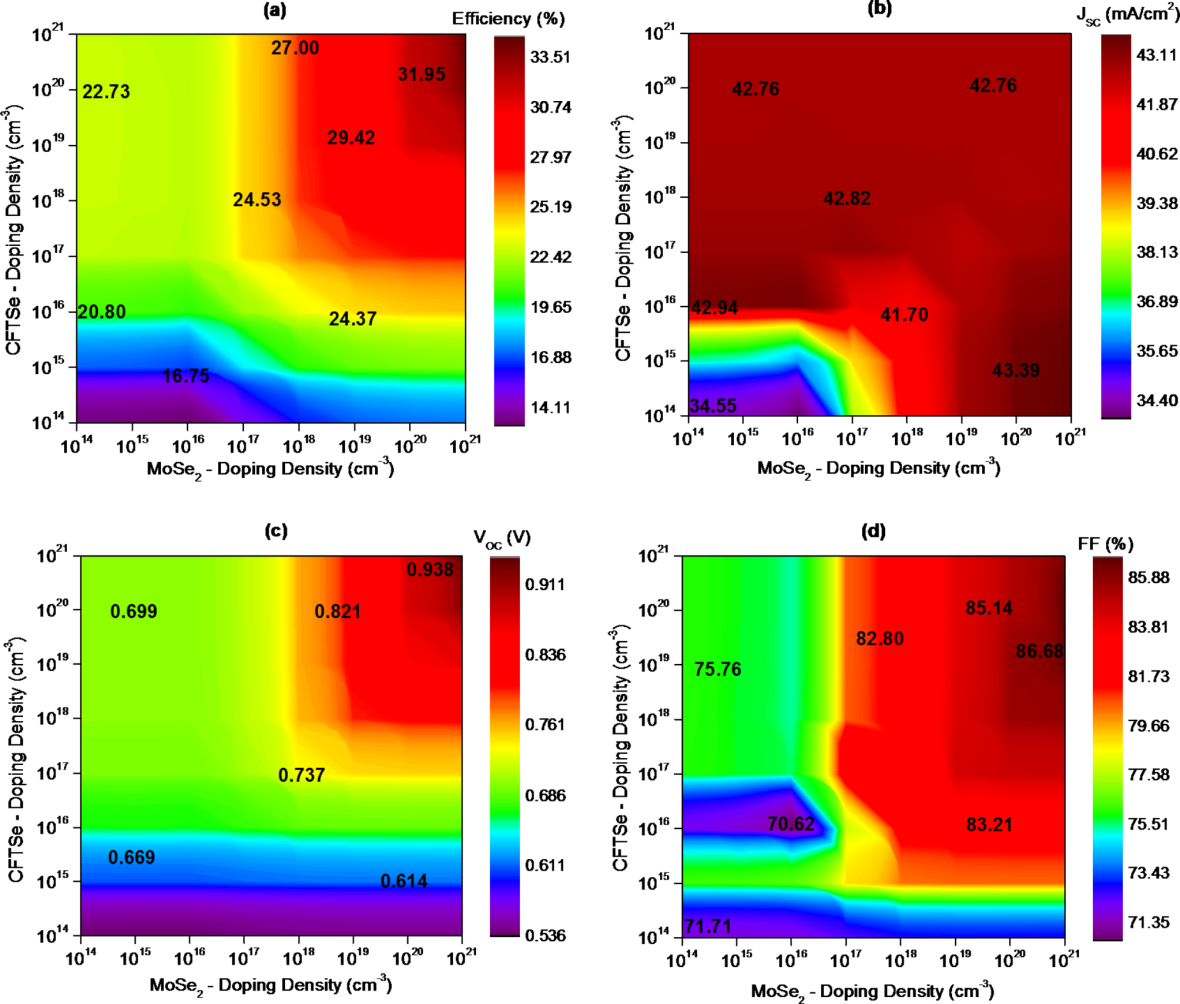
The impact of CFTSe doping density on the photovoltaic performance of CFTSe-based solar cells.

Doping in CFTSe and MoSe_2_ can significantly improve their electronic properties, tunability, and ultimately their impact on overall device performance. Experimentally, doping densities in the range of 10^20^ cm^−3^ to 10^21^ cm^−3^ can often be achieved through techniques like co-evaporation or chemical vapor deposition (CVD). For instance, high doping densities in materials similar to CFTSe, such as CZTSe, have been reported. S. Giraldo et al. demonstrated an In-doped CZTSe thin film with a doping density of 2.6 × 10^20^ cm^−3^^63^. In MoSe_2_, doping can be experimentally controlled using methods like chemical vapor deposition, ion implantation, or post-synthesis annealing. Several studies have shown that doping densities in MoSe_2_ can be fine-tuned by incorporating dopants during the growth process. For example, doping can be achieved using precursors containing elements such as phosphorus or copper, or by bombarding MoSe_2_ with ions of dopant materials like nitrogen (for n-type) or aluminum (for p-type). M. Li et al. reported p-type large-area monolayers of MoS_2_ via Nb-doping using the chemical vapor deposition method^64^. Similarly, S. Prucnal et al. demonstrated n-type doping in MoSe_2_ flakes through chlorine ion implantation followed by annealing^65^. Furthermore, various studies have explored the formation and electrical tunability of doped MoSe_2_. Researchers have reported adjusting the electrical conductivity of MoS_2_ monolayers through substitutional doping, with tunable concentrations of Nb and Re, using metal-organic chemical vapor deposition (MOCVD) techniques^66^. These advancements highlight the versatility of doping in improving the electronic performance of CFTSe and MoSe_2_ for solar cell applications. Figure 4 illustrates the impact of doping concentration within the absorber layer on various solar cell performance parameters. The doping concentration varied from 1 × 10^14^ cm^− 3^ to 1 × 10^21^ cm^− 3^ for both the CFTSe and MoSe_2_ layers. The figure demonstrates a clear correlation between device performance and the carrier concentration of the CFTSe absorber and MoSe_2_ buffer layer. For example, as the CFTSe doping density increases from 10^14^ cm^− 3^ to 10^21^ cm^− 3^ at a 10^14^ cm^− 3^ doping density of MoSe_2_, J_sc_ rises from 34.55 mA/cm^2^ to 42.765 mA/cm^2^, PCE increases from 13.33 to 22.73%, V_oc_ improves from 0.538 V to 0.699 V and FF enhances from 71.71 to 76%. Beyond a CFTSe doping density of 10^18^ cm^− 3^, the J_sc_ does not exhibit significant variation with changes in MoSe_2_ doping density. For instance, J_sc_ is 42.82 mA/cm^2^ at a CFTSe doping density of 10^18^ cm^− 3^ and 42.76 mA/cm^2^ at 10^20^ cm^− 3^, CFTSe doping density, respectively, as shown in Fig. 4 (b). This behavior can be attributed to the reduction in the lifetime of photogenerated electrons associated with the rise in carrier concentration, which lowers the possibilities of carrier collection, resulting in a lower J_sc_. Optimal performance is achieved with both MoSe_2_ and CFTSe doping density of 10^21^ cm^− 3^, resulting in an efficiency of 34.53%, J_sc_ of 42.76 mA/cm^2^, V_oc_ of 0.938 V, and FF of 86.11%. It is noteworthy that high doping densities in MoSe_2_ have been experimentally achieved up to approximately 3 × 10^19^ cm^−3^, as reported by J. Suh et al.^66^. However, reaching doping levels as high as 10^21^ cm^−3^ presents a significant experimental challenge. In our simulation, we investigated the impact of high doping levels in both MoSe_2_ and CFTSe on device performance. This exploration offers valuable insights and could inspire future experimental studies aimed at achieving these higher doping concentrations to further enhance device efficiency.

The observed results can be attributed to the reduction in the reverse saturation current J_o_ as the absorber carrier concentration increases. Since the V_oc_ is inversely proportional to the J_o_, a decrease in J_o_ results in a higher V_oc_. This increase in V_oc_ enhances the overall conversion efficiency of the solar cells, represented by the formula: $\:\eta\:={\text{V}}_{oc}\:{\text{J}}_{\text{s}\text{c}}\:FF/{\text{P}}_{\text{i}\text{n}}.\:$Thus, reducing J_o_ and increasing V_oc_ leads to improved solar cell efficiency. Additionally, considering the depletion width *W*_d_ of a p-n heterojunction and its impact on the photogenerated current^67–69^. The photogenerated current is directly proportional to *W*_d_, which is primarily influenced by the carrier concentration. A wider W_d_ leads to better carrier collection, resulting in more photogenerated current and, consequently, a higher V_oc_. This effect can be understood through field-effect theory, which explains how the electric field extends to the edges of the depletion region, effectively attracting photogenerated electrons into the buffer layer. Regarding the buffer layer, a highly doped layer facilitates a beneficial downward bending of the conduction band at the interface, which improves the collection of photoelectrons^70^. Higher carrier density in the buffer layer allows for greater ionization of atoms and, consequently, more photogenerated electrons. The reduction in the electrostatic potential barrier at the buffer/absorber and window/buffer heterojunctions expands the space charge region. This expansion enhances the collection of photogenerated carriers, thereby boosting the conversion efficiency.

**Fig. 5:**
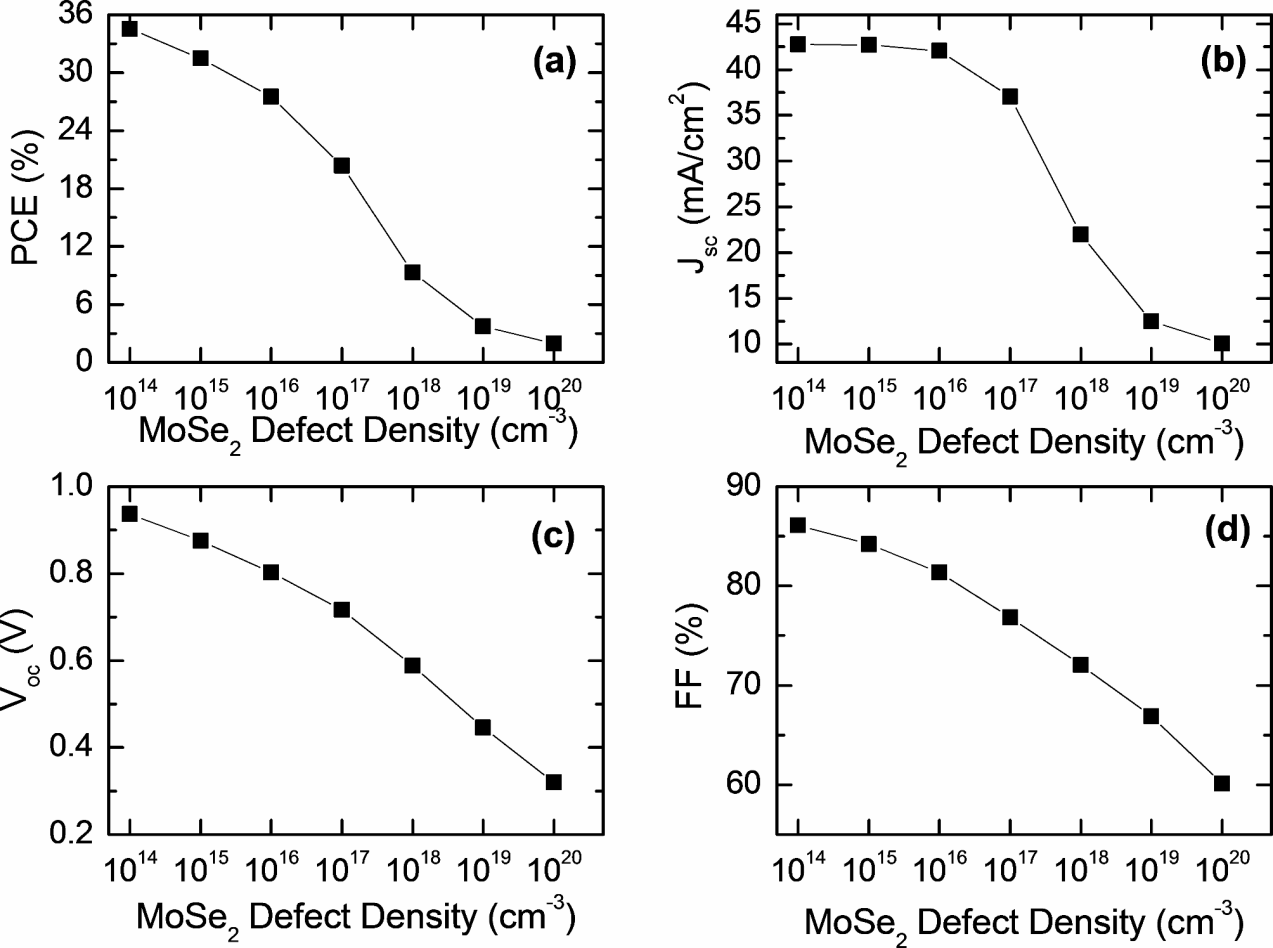
Effect of MoSe_2_ defect density on the photovoltaic performance (**a**) PCE, (**b**) J_SC_, (**c**) V_OC_, and (**d**) FF of CFTSe-based solar cells.

**Fig. 6:**
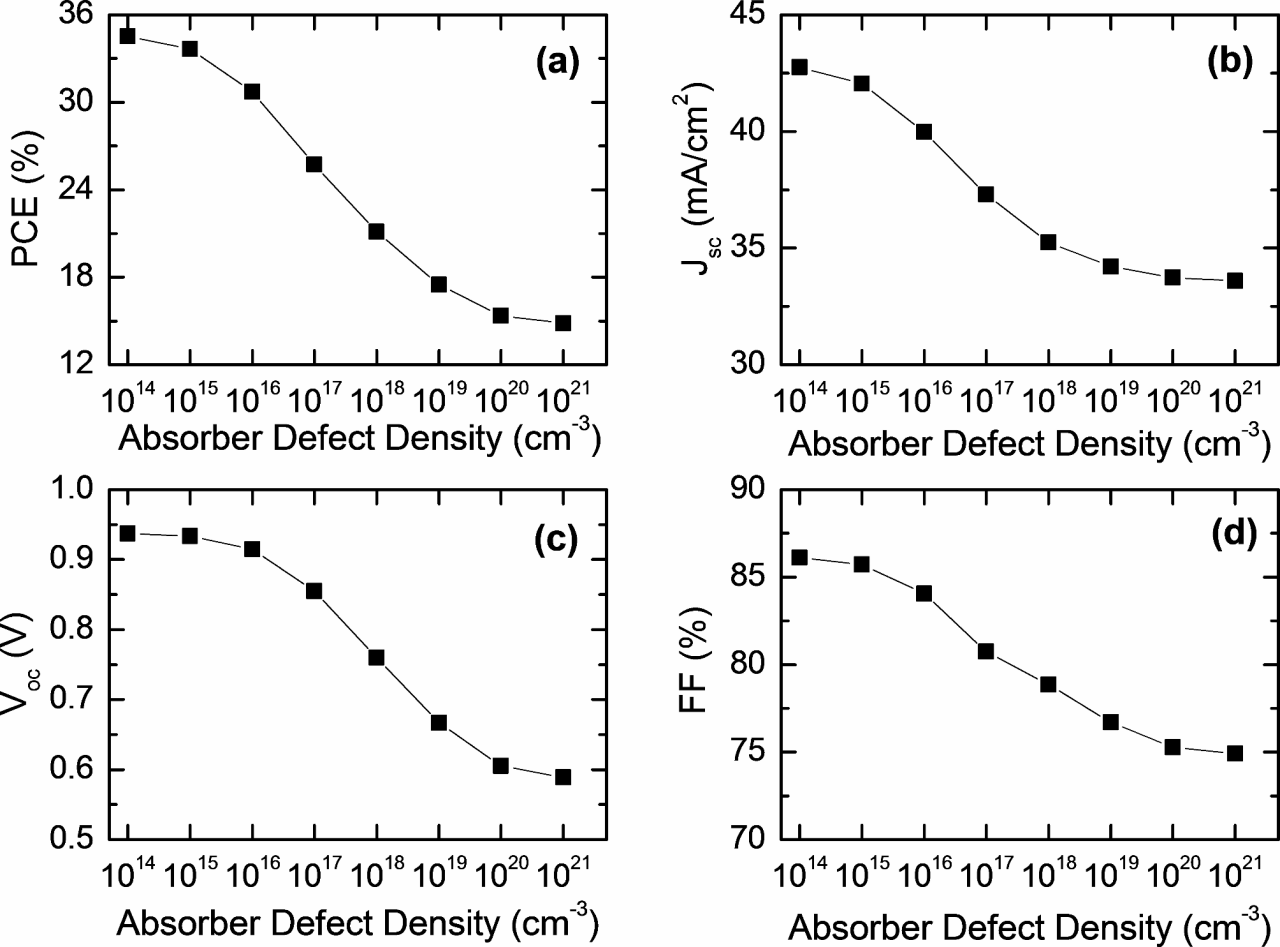
Effect of CFTSe defect density on the photovoltaic performance (**a**) PCE, (**b**) J_SC_, (**c**) V_OC_, and (**d**) FF of CFTSe-based solar cells.

Defect states in bulk materials play a crucial role in shaping the optoelectronic properties of thin-film semiconductors by serving as recombination sites for photogenerated carriers. We propose that the MoSe_2_ layer effectively mitigates surface defects, enhancing photon absorption and reducing recombination losses in both the absorber layer and the absorber-buffer interface. Previous studies on similar TMDC materials, such as MoS_2_ in quaternary semiconductors, have demonstrated its ability to extrinsically passivate grain boundaries (GBs) within the absorber layer^49^. As a result, passivated GBs no longer serve as high-recombination sites for minority carriers and act as efficient current pathways for minority carrier collection, improving overall device performance. Figures 5 and 6 demonstrate the impact of defect densities on the photovoltaic parameters of both the absorber and interfacial layers. For the MoSe_2_ buffer layer, defect densities ranged from 10^14^ cm^− 3^ to 10^20^ cm^− 3^ while for the absorber layer, they ranged up to 10^21^ cm^− 3^. Figure 5 shows that increasing defect density adversely affects photovoltaic parameters such as PCE, Jsc, FF, and V_oc_. Specifically, as defect density rises from 10^14^ cm^− 3^ to 10^20^ cm^− 3^, PCE drops from 34.53 to 1.94%, and J_sc_, V_oc_, and FF decrease from 42.76 mA/cm^2^, 0.937 V, and 86.11% to 10.07 10.07 mA/cm^2^, 0.320 V, and 60.11%, respectively. Figure 6 further illustrates that as defect density increases from 10^14^ cm^− 3^ to 10^21^ cm^− 3^, PCE, J_sc_, V_oc_, and FF decline from 34.53%, 42.76 mA/cm^2^, 0.937 V, and 86.11–14.85%, 33.60 mA/cm^2^, 0.589 V, and 74.92%, respectively.

To fully grasp the results, it’s worth considering the significant impact of defect states on the performance of kieserite quaternary solar cells. These defects serve as trapping sites for photogenerated electron-hole pairs, which can adversely affect the efficiency and overall performance of the solar cells. They hinder the effective collection of charge carriers at both the front and back contacts, leading to increased recombination rates and, consequently, a degradation in device performance^71–73^. An increase in defect density results in higher saturation levels, which modifies the band gap structure of the absorber layers and disrupts the absorption process, causing a notable decrease in J_sc_. As defect concentration rises, more trapping centers are created for photogenerated charge carriers, preventing their effective transport to the back contacts and resulting in a significant drop in device performance. Therefore, high-quality active layers are crucial for future advancements in solar cell technology. Given that the CFTSe absorber has a lower activation energy than its bandgap, the increased defect density at deeper defect levels contributes to recombination loss. This is primarily attributed to dominant recombination at the buffer/absorber interface, along with other recombination events^73,74^. When defect density rises, the minority carrier lifetime decreases, resulting in a shorter diffusion length for electrons and holes within the absorber layer, which in turn leads to an increase in recombination losses.

An effective method for reducing defect density is meticulous management during the fabrication process. Although various techniques for minimizing defects have been proposed, many of these methods can increase fabrication costs. Alternatively, technological modifications, such as incorporating nanostructures like quantum wells and dots, offer a promising solution. This approach involves stacking multiple layers of nanometer-thick wells and barrier layers within the absorber layer^75–77^. Due to the extremely thin layers of absorber material, defect density is significantly reduced or even negligible, resulting in lower recombination rates and enhanced device performance.

**Fig. 7:**
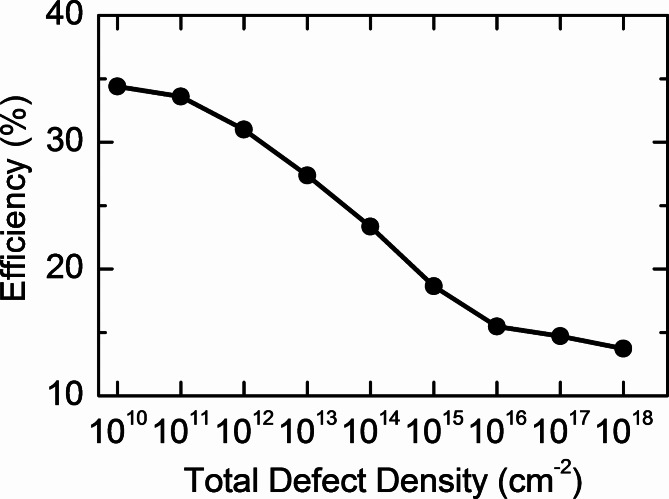
The effect of the back interface defect density on the solar cell efficiency.

For a more in-depth investigation, the impact of interface defect density on solar cell performance was analyzed. Typically, high defect densities at the interface can scatter charge carriers, resulting in lower carrier mobility and increased resistivity. These defects also act as traps, heightening recombination rates and adversely affecting the overall efficiency of optoelectronic devices. Furthermore, interface defects can introduce mid-gap states, disrupting both absorption and emission processes, which adversely affects light generation and detection efficiency^78,79^. As shown in Fig. 7, increasing the interface defect density at the MoSe_2_/CFTSe junction from 10^10^ cm⁻^2^ to 10^18^ cm⁻^2^ dramatically reduces the solar cell efficiency from 34.39 to 13.71%. This significant efficiency drop indicates rapid recombination at the interface, likely caused by the formation of intermediate energy levels between the layers.

**Fig. 8:**
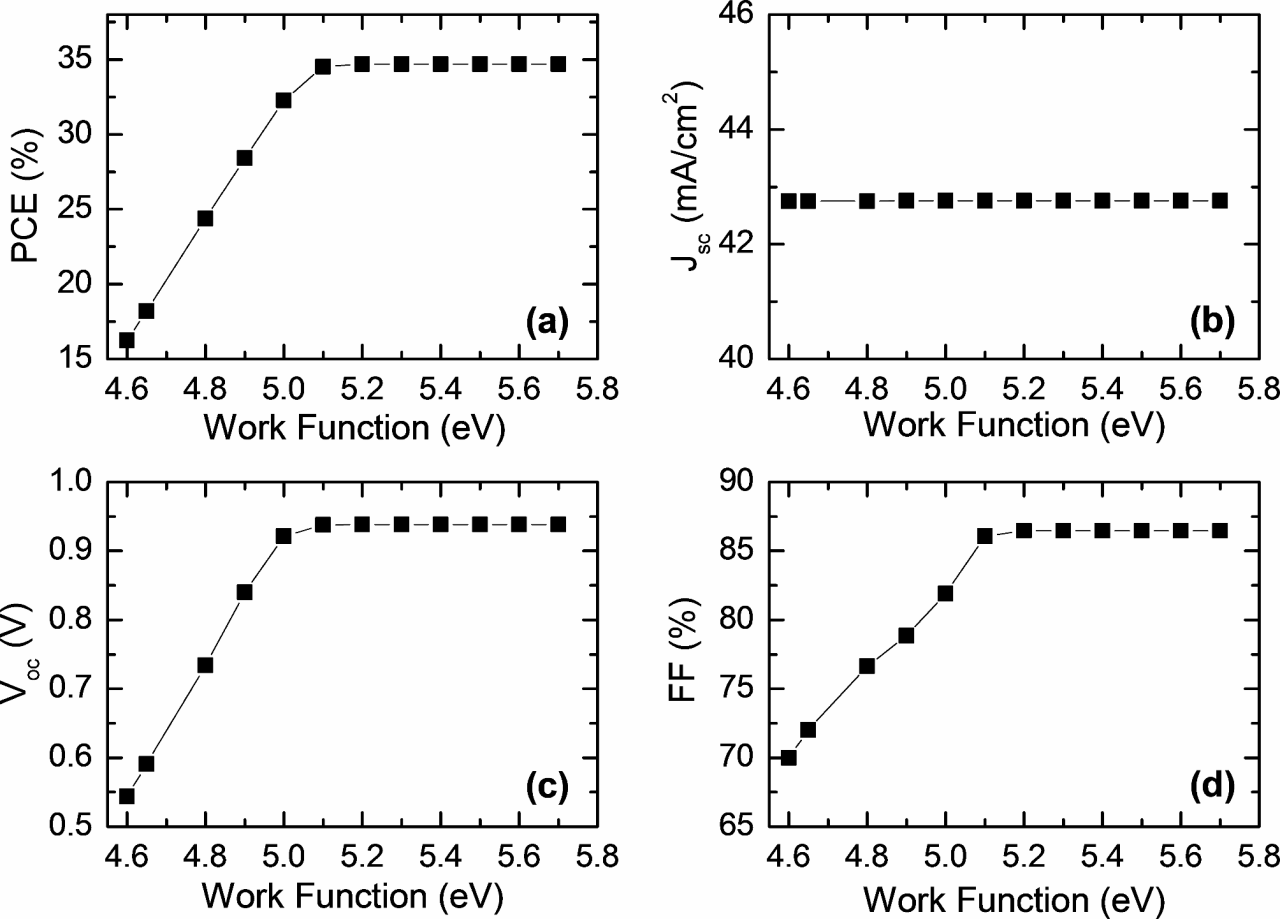
The effect of the back contact work function on the photovoltaic performance of CFTSe-based solar cells, including (**a**) PCE, (**b**) J_sc_, (**c**) V_oc_, and (**d**) FF.

The work function Φ_m_ of the back metal contact is a critical factor that significantly influences a solar cell’s performance^80,81^. In our study, Mo is utilized as a back contact, with a work function of 5.1 eV. To further explore the impact of this factor, we conducted an investigation examining how variations in the back metal contact’s work function affect the cell’s electrical output. The results are illustrated in Fig. 8. The study varied the work function of the back metal contact between 4.6 eV and 5.7 eV. For work functions ranging from 4.6 eV to 5.1 eV, significant changes in the solar cell’s output parameters were observed, though the short-circuit current density (J_sc_) remained unchanged. Beyond a work function 5.1 eV, no additional variations in the parameters were noted. The best performance was achieved with a back contact work function of 5.1 eV, resulting in an efficiency of 34.51%, J_sc_ of 42.76 mA/cm^2^, V_oc_ of 0.938 V, and a FF of 86.07%. The results can be explained within the framework of the Schottky barrier and ohmic-nature formation at the interface contact. In our model, the adjacent layer to the back contact is a p-type. Thus, the metal work function Φ_m_ is expressed as^82^:

2 $$\:{\varPhi\:}_{m}=\chi\:+{E}_{g}-{k}_{B}T\text{ln}\left(\frac{{N}_{C}}{{N}_{A}-{N}_{D}}\right)$$

where $\:\chi\:$ is the electron affinity, $\:{E}_{g}$ is the energy band gap. $\:{N}_{C}$ is the conduction band effective density of states. $\:{N}_{A}$, and $\:{N}_{D}$ are the acceptors and donors concentrations, respectively. When the metal work function, Φ_m_, is significantly greater than that of the absorber layer, the junction acts as an ohmic contact, enhancing the efficiency of the solar cell. Conversely, if the metal work function is low, Schottky behavior prevails at the junction, leading to reduced performance of the solar cell. For perovskites, the work function varies depending on the specific type of perovskite, its composition, synthesis, and processing methods used. In general, a high metal work function above 5 eV is beneficial to realize an ohmic behavior.

**Fig. 9:**
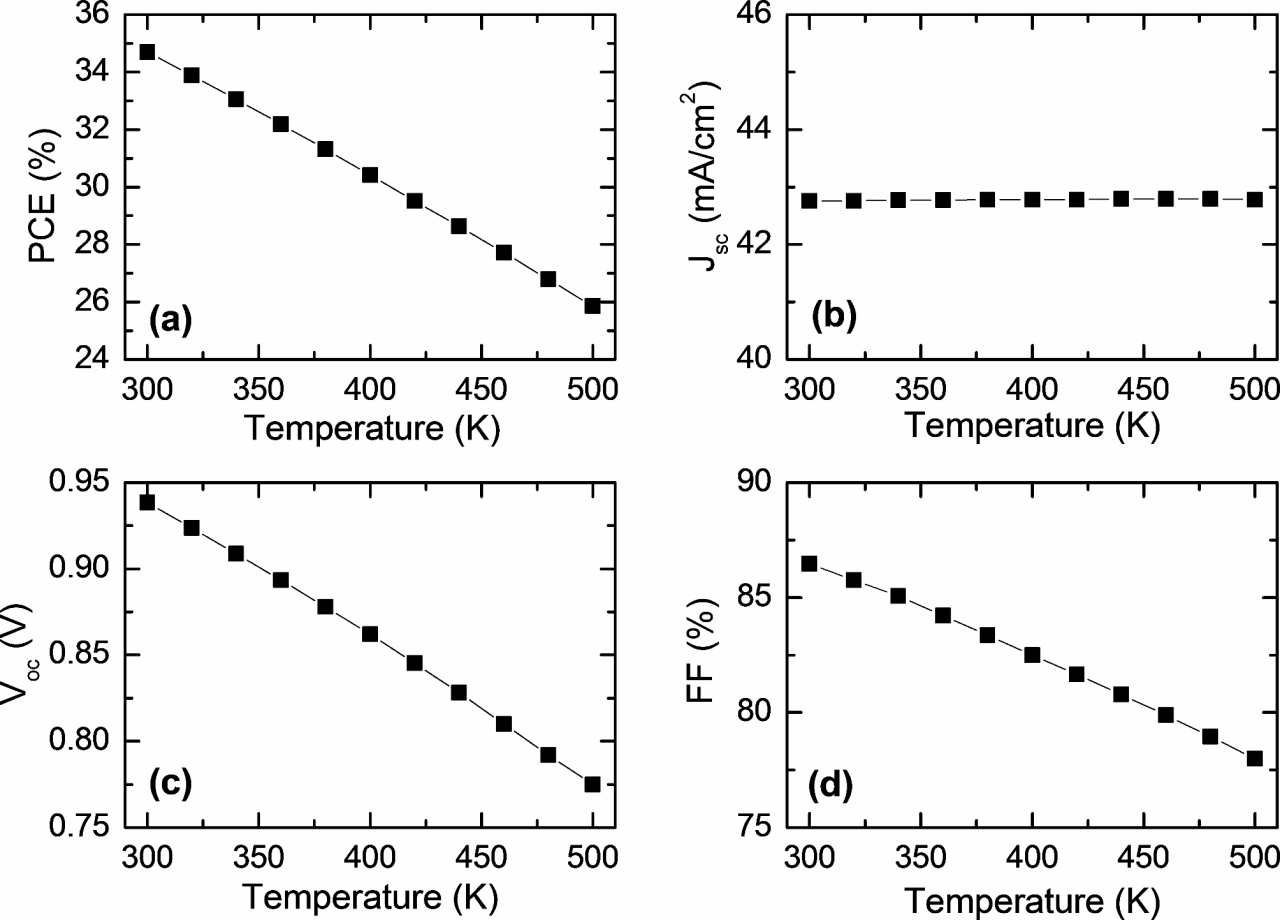
Effect of operating temperature on the photovoltaic performance (**a**) PCE (**b**) J_SC_, (**c**) V_OC_, and (**d**) FF of CFTSE-based solar cell.

Finally, the impact of the device’s operating temperature on its output parameters was examined across a temperature range from 300 K to 500 K. The results are shown in Fig. 9. At 500 K, the parameters are as follows: Efficiency 25.86%, J_sc_ 42.80 mA/cm^2^, V_oc_ 0.775 V, and FF 78%. In contrast, at 300 K, the optimal parameters are Efficiency 34.69%, J_sc_ 42.76 mA/cm^2^, V_oc_ 0.938 V, and FF 86.47%. While J_sc_ remains nearly constant with increasing temperature, the V_oc_, FF and the PCE decrease as the temperature rises, indicating a negative impact on the solar cell’s performance. In a solar cell, the parameter most influenced by rising temperatures is the open-circuit voltage (V_oc_). A decrease in the V_oc_ can be seen as an increase in the dark current. This effect is primarily due to the narrowing of the band gap as temperature rises. Higher temperatures reduce the band gap, which speeds up the recombination of electrons and holes between the conduction and valence bands^83^. This increased recombination rate raises the dark current within the cell. As temperature increases, electrons acquire more thermal energy, leading to more frequent recombination with holes before they reach the depletion region and are collected. The reduction in the PCE with higher temperatures is largely due to the decreased V_oc_. The narrowing band gap at elevated temperatures accelerates electron-hole recombination, resulting in increased dark current and, consequently, diminished cell performance^84^.

**Conclusions**.

Among the quaternary chalcogenides based on copper, Cu_2_FeSnSe_4_ (CFTSe) stands out as a promising yet under-explored material for solar cell applications. This study investigates a novel heterostructure solar cell that utilizes CFTSe as the absorbing layer and introduces MoSe2 TMDC as an innovative buffer layer, employing SCAPS simulation for analysis. The research includes the optimization of the absorber layer, followed by an in-depth investigation of the new buffer layer. Key photovoltaic parameters such as J_sc_, V_oc_, FF, and PCE are analyzed concerning the thickness, doping, defect density, and work function of the structure. Our findings reveal that incorporating the MoSe_2_ layer between the absorber and the molybdenum back contact significantly enhances the device’s overall performance by facilitating the formation of ohmic contacts. The optimized parameters result in a remarkable PCE of 34.69%, a J_sc_ of 42.76 mA/cm^2^, a V_oc_ of 0.938 V, and a FF of 86.47%. Additionally, we explore the impact of operating temperature on cell performance over the range of 300 to 500 K. The observed decline in V_oc_ with increasing temperature is attributed to the rise in saturation current. This study highlights the untapped potential of CFTSe as a promising absorber material and underscores that the proposed TMDC MoSe_2_ buffer layer presents an emerging alternative for achieving superior performance parameters.

## Data Availability

The datasets used and/or analysed during the current study available from the corresponding author on reasonable request.

## References

[1] Wolden, C. A. et al. Photovoltaic manufacturing: Present status, future prospects, and research needs. *J. Vacuum Sci. Technol. A: Vacuum Surf. Films* **29**, (2011).

[2] Sk, M. Recent progress of lead-free halide double perovskites for green energy and other applications. *Appl. Phys. A*. **128**, 462 (2022).

[3] Gourav, Sk, M., Ramachandran, K. & Ghosh, S. First-principles investigation of Rb_2_ Ag (Ga/In)Br _6_ for thermoelectric and photovoltaic applications. *Int. J. Quantum Chem.* **122**, (2022).

[4] Sk, M. & Ghosh, S. First-principles investigation of structural, optoelectronic, and thermoelectric properties of Cs _2_ tl(As/Sb)I _6_. *Int. J. Energy Res.* **46**, 10553–10563 (2022).

[5] Jackson, P. et al. Properties of Cu(In,Ga)Se _2_ solar cells with new record efficiencies up to 21.7%. *Phys. Status Solidi (RRL) Rapid Res. Lett.* **9**, 28–31 (2015).

[6] Zhou, J., Chen, X., Wang, Y. & Zhao, B. Influence of glass frits on the formation of back surface field in silicon solar cell. *Mater. Lett.* **169**, 197–199 (2016).

[7] Tang, K. et al. Stoichiometry dependence of the optical and minority-carrier lifetime behaviors of CdTe epitaxial films: a low-temperature and time-resolved photoluminescence study. *Appl. Surf. Sci.* **387**, 477–482 (2016).

[8] Meng, X. et al. Synthesis of Cu2FeSnSe4 thin film by selenization of RF Magnetron sputtered precursor. *Mater. Lett.* **117**, 1–3 (2014).

[9] Jackson, P. et al. Effects of heavy alkali elements in Cu(in,Ga)Se _2_ solar cells with efficiencies up to 22.6%. *Physica status solidi (RRL) –*. *Rapid Res. Lett.* **10**, 583–586 (2016).

[10] Gohri, S., Madan, J. & Pandey, R. Augmenting CIGS Solar Cell Efficiency through multiple Grading Profile Analysis. *J. Electron. Mater.* **52**, 6335–6349 (2023).

[11] Yakushev, M. V., Faugeras, C., Mudryi, A. V. & Martin, R. W. A Magneto-Reflectivity Study of CuInTe _2_ single crystals. *Phys. Status Solidi (b)* 257, (2020).

[12] Song, T., Kanevce, A. & Sites, J. R. Emitter/absorber interface of CdTe solar cells. *J. Appl. Phys.* **119**, (2016).

[13] Hernández-Calderón, V. et al. CdS/ZnS Bilayer Thin films used as buffer layer in 10%-Efficient cu _2_ ZnSnSe _4_ solar cells. *ACS Appl. Energy Mater.* **3**, 6815–6823 (2020).

[14] Tan, X., Hu, J., Zhu, W., Wu, F. & Han, X. Improved performance of kesterite Cu2ZnSn(S,Se)4 thin film solar cells by Ag/Ge co-doping. *J. Alloys Compd.* **981**, 173645 (2024).

[15] Yadav, A., Patel, A. K. & Mishra, R. Modeling and Simulation of CZTS Solar cell using zn _1-x_ mg _x_ O as a buffer layer and cul as a hole transport layer for efficiency improvement. *Eng. Res. Express*. **6**, 015031 (2024).

[16] He, M. et al. Kesterite Solar cells: insights into current strategies and challenges. *Adv. Sci.* **8**, (2021). 10.1002/advs.202004313 PMC8097387 33977066

[17] Moustafa, M., Mourched, B., Salem, S. & Yasin, S. Performance enhancement of CZTS-based solar cells with tungsten disulfide as a new buffer layer. *Solid State Commun.* **359**, 115007 (2023).

[18] Wang, W. et al. Device characteristics of CZTSSe Thin-Film Solar cells with 12.6% efficiency. *Adv. Energy Mater.* **4**, (2014).

[19] Gohri, S., Madan, J. & Pandey, R. Optimizing photovoltaic performance: Strategic enhancement of Ag-doped graded CAZTS solar cells achieving 27.3% efficiency. *Inorg. Chem. Commun.* **163**, 112394 (2024).

[20] Suryawanshi, M. P. et al. CZTS based thin film solar cells: a status review. *Mater. Technol.* **28**, 98–109 (2013).

[21] Zhou, J., Hu, Y. & Chen, X. Preparation of photovoltaic absorption material Cu2FeSnSe4 microparticles via an atmospheric pressure liquid reflux method. *Mater. Lett.* **184**, 216–218 (2016).

[22] Meng, X. et al. Synthesis and characterization of Cu-based selenide photovoltaic materials: Cu2FeSnSe4 and Cu(in, Al)Se2. *J. Alloys Compd.* **644**, 354–362 (2015).

[23] Nefzi, C., Souli, M., Cuminal, Y. & Kamoun-Turki, N. Effect of sulfur concentration on structural, optical and electrical properties of Cu2FeSnS4 thin films for solar cells and photocatalysis applications. *Superlattices Microstruct.* **124**, 17–29 (2018).

[24] Tripathi, S., Srivastva, R., Kumar, B. & Dwivedi, D. K. Deposition and characterization of stannite Cu2FeSn(S0·8Se0.2)4 thin film for potential absorber layer in solar cell application. *Opt. Mater. (Amst)*. **120**, 111430 (2021).

[25] Bhattacharya, R. N. et al. High efficiency thin-film CuIn1–xGaxSe2 photovoltaic cells using a Cd1–xZnxS buffer layer. *Appl. Phys. Lett.* **89**, (2006).

[26] Moradi, M., Teimouri, R., Saadat, M. & Zahedifar, M. Buffer layer replacement: a method for increasing the conversion efficiency of CIGS thin film solar cells. *Optik (Stuttg)*. **136**, 222–227 (2017).

[27] Belarbi, F., Rahal, W., Rached, D., benghabrit, S. & Adnane, M. A comparative study of different buffer layers for CZTS solar cell using Scaps-1D simulation program. *Optik (Stuttg)*. **216**, 164743 (2020).

[28] Tousif, M. N., Mohamma, S., Ferdous, A. A. & Hoque Md. A. Investigation of different materials as buffer layer in CZTS Solar cells using SCAPS. *J. Clean. Energy Technol.* **6**, 293–296 (2018).

[29] Bouarissa, A., Gueddim, A., Bouarissa, N. & Maghraoui-Meherezi, H. Modeling of ZnO/MoS2/CZTS photovoltaic solar cell through window, buffer and absorber layers optimization. *Mater. Sci. Eng. B*. **263**, 114816 (2021).

[30] Ravindra, N. M., Tang, W. & Rassay, S. Transition Metal Dichalcogenides Properties and Applications. in *Semiconductors* 333–396 (Springer International Publishing, Cham, 2019). 10.1007/978-3-030-02171-9_6.

[31] Manzeli, S., Ovchinnikov, D., Pasquier, D., Yazyev, O. V. & Kis A. 2D transition metal dichalcogenides. *Nat. Rev. Mater.* **2**, 17033 (2017).

[32] Moustafa, M., Paulheim, A., Mohamed, M., Janowitz, C. & Manzke, R. Angle-resolved photoemission studies of the valence bands of ZrS Se2–. *Appl. Surf. Sci.* **366**, 397–403 (2016).

[33] Wang, Q. H., Kalantar-Zadeh, K., Kis, A., Coleman, J. N. & Strano, M. S. Electronics and optoelectronics of two-dimensional transition metal dichalcogenides. *Nat. Nanotechnol*. **7**, 699–712 (2012). 23132225 10.1038/nnano.2012.193

[34] Böker, T. et al. Band structure of MoS2, MoSe2, and 훼 –MoTe2 : Angle-resolved photoelectron spectroscopy and ab initio calculations. *Phys. Rev. B*. **64**, 235305 (2001).

[35] Moustafa, M., Ghafari, A., Paulheim, A., Janowitz, C. & Manzke, R. Spin orbit splitting in the valence bands of ZrSxSe2–x: Angle resolved photoemission and density functional theory. *J. Electron. Spectros Relat. Phenom.* **189**, 35–39 (2013).

[36] Wang, H., Yuan, H., Sae Hong, S., Li, Y. & Cui, Y. Physical and chemical tuning of two-dimensional transition metal dichalcogenides. *Chem. Soc. Rev.* **44**, 2664–2680 (2015). 25474482 10.1039/c4cs00287c

[37] Voiry, D. et al. Enhanced catalytic activity in strained chemically exfoliated WS2 nanosheets for hydrogen evolution. *Nat. Mater.* **12**, 850–855 (2013). 23832127 10.1038/nmat3700

[38] Jain, A. et al. Commentary: the materials project: a materials genome approach to accelerating materials innovation. *APL Mater.* **1**, (2013).

[39] Finn, S. T. & Macdonald, J. E. Contact and support considerations in the hydrogen evolution reaction activity of Petaled MoS _2_ electrodes. *ACS Appl. Mater. Interfaces*. **8**, 25185–25192 (2016). 27564136 10.1021/acsami.6b05101

[40] Zhao, W. et al. Origin of Indirect Optical transitions in few-layer MoS _2_, WS _2_, and WSe _2_. *Nano Lett.* **13**, 5627–5634 (2013). 24168432 10.1021/nl403270k

[41] Burgelman, M., Decock, K., Khelifi, S. & Abass, A. Advanced electrical simulation of thin film solar cells. *Thin Solid Films*. **535**, 296–301 (2013).

[42] Burgelman, M., Nollet, P. & Degrave, S. Modelling polycrystalline semiconductor solar cells. *Thin Solid Films*. **361–362**, 527–532 (2000).

[43] Sk, M. & Ghosh, S. 16.35% efficient Cs2GeSnCl6 based heterojunction solar cell with hole-blocking SnO2 layer: DFT and SCAPS-1D simulation. *Optik (Stuttg)*. **267**, 169608 (2022).

[44] Moustafa, M., Zoubi, T., Al & Yasin, S. Numerical analysis of the role of p-MoSe2 interfacial layer in CZTSe thin-film solar cells using SCAPS simulation. *Optik (Stuttg)*. **247**, 167885 (2021).

[45] AlZoubi, T., Moghrabi, A., Moustafa, M. & Yasin, S. Efficiency boost of CZTS solar cells based on double-absorber architecture: device modeling and analysis. *Sol. Energy*. **225**, 44–52 (2021).

[46] Moustafa, M. & AlZoubi, T. Effect of the n-MoTe2 interfacial layer in cadmium telluride solar cells using SCAPS. *Optik (Stuttg)*. **170**, 101–105 (2018).

[47] Baro, M. & Borgohain, P. SCAPS-1D device simulation of highly efficient perovskite solar cells using diverse charge transport layers. *J. Electron. Mater.* **52**, 7623–7644 (2023).

[48] Shafayet-Ul-Islam, M., Kuddus, A., Kabiruzzaman, M., Farhad, S. F. U. & Kowsar, A. Cu2FeSnS4-based heterojunction solar cells with M O (M=Cu, Ni)-back surface field layers: impact of defect density states and recombination. *Next Energy*. **6**, 100196 (2025).

[49] Yang, K. et al. Effects of na and MoS _2_ on Cu _2_ ZnSnS _4_ thin-film solar cell. *Prog. Photovoltaics Res. Appl.* **23**, 862–873 (2015).

[50] Khadka, D. B. & Kim, J. Structural, optical and electrical properties of Cu2FeSnX4 (X=S, Se) thin films prepared by chemical spray pyrolysis. *J. Alloys Compd.* **638**, 103–108 (2015).

[51] Konan, F. K. Hervé Joël Tchognia Nkuissi & Bouchaib Hartiti. Numerical simulations of highly efficient Cu2FeSnS4 (CFTS)-based solar cells. *Int. J. Renew. Energy Res.*10.20508/ijrer.v9i4.9816.g7829 (2019).

[52] Ali, M. H. et al. Performance enhancement of an MoS _2_ -Based Heterojunction Solar cell with an in _2_ Te _3_ back Surface Field: A Numerical Simulation Approach. *ACS Omega*. **8**, 7017–7029 (2023). 36844558 10.1021/acsomega.2c07846 PMC9948157

[53] Atowar Rahman, M. Enhancing the photovoltaic performance of Cd-free Cu2ZnSnS4 heterojunction solar cells using SnS HTL and TiO2 ETL. *Sol. Energy*. **215**, 64–76 (2021).

[54] Meng, X. et al. Structural, optical and electrical properties of Cu2FeSnSe4 and Cu(in,Al)Se2 thin films. *Mater. Sci. Semicond. Process.* **39**, 243–250 (2015).

[55] Riyad, M. N. H. et al. Performance evaluation of WS_2_ as buffer and Sb_2_ S_3_ as hole transport layer in CZTS solar cell by numerical simulation. *Eng. Rep.* **5**, (2023).

[56] Sedhain, R. P., & Kaphle, G. C. Structural and electronic properties of transition metal di-chalcogenides (MX2) M=(Mo, W) AND X=(S, Se) in bulk state: a first-principles study. *J. Inst. Sci. Technol.* **22**, 41–50 (2017).

[57] Sun, Y. et al. Defect Control for High-Efficiency Cu _2_ ZnSn(S,Se) _4_ solar cells by atomic layer deposition of Al _2_ O _3_ on Precursor Film. *Solar RRL* **5**, (2021).

[58] Moon, E., Barrow, M., Lim, J., Blaauw, D. & Phillips, J. D. Dual-Junction GaAs Photovoltaics for Low Irradiance Wireless Power Transfer in submillimeter-scale Sensor Nodes. *IEEE J. Photovolt.* **10**, 1721–1726 (2020). 33224555 10.1109/JPHOTOV.2020.3025450 PMC7675925

[59] Cheng, J. L. et al. Ductile Zr-Based Bulk Metallic glasses by Controlling Heterogeneous microstructure from Phase Competition Strategy. *Nanomaterials* **9**, 1728 (2019). 31817045 10.3390/nano9121728 PMC6955685

[60] Chelvanathan, P., Hossain, M. I. & Amin, N. Performance analysis of copper–indium–gallium–diselenide (CIGS) solar cells with various buffer layers by SCAPS. *Curr. Appl. Phys.* **10**, S387–S391 (2010).

[61] Lin, P. et al. Numerical simulation of Cu2ZnSnS4 based solar cells with In2S3 buffer layers by SCAPS-1D. *J. Appl. Sci. Eng.* **17**, 383–390 (2014).

[62] Sarkar, K. Effects of very thin CdS window layer on CdTe solar cell. *J. Mech. Continua Math. Sci.* **14**, 14–29 (2019).

[63] Giraldo, S. et al. Optical and electrical properties of In-doped Cu2ZnSnSe4. *Sol. Energy Mater. Sol. Cells*. **151**, 44–51 (2016).

[64] Li, M. et al. P-type doping in large-area monolayer MoS _2_ by Chemical Vapor Deposition. *ACS Appl. Mater. Interfaces*. **12**, 6276–6282 (2020). 31937099 10.1021/acsami.9b19864

[65] Prucnal, S. et al. Chlorine doping of MoSe _2_ flakes by ion implantation. *Nanoscale* **13**, 5834–5846 (2021). 33720250 10.1039/d0nr08935d

[66] Gao, H. et al. Tuning Electrical Conductance of MoS _2_ Monolayers through Substitutional Doping. *Nano Lett.* **20**, 4095–4101 (2020). 32396734 10.1021/acs.nanolett.9b05247

[67] Suh, J. et al. Doping against the native propensity of MoS _2_ : Degenerate Hole Doping by Cation Substitution. *Nano Lett.* **14**, 6976–6982 (2014). 25420217 10.1021/nl503251h

[68] Li, S. S. Photonic devices. in Semiconductor Physical Electronics 327–390 (Springer US, Boston, MA, doi:10.1007/978-1-4613-0489-0_12. (1993).

[69] Green, M. A. Solar cells: operating principles, technology, and system applications. *Englewood Cliffs* (1982).

[70] Liao, D. & Rockett, A. Cd doping at the CuInSe2/CdS heterojunction. *J. Appl. Phys.* **93**, 9380–9382 (2003).

[71] Chen, S., Yang, J. H., Gong, X. G., Walsh, A. & Wei, S. H. Intrinsic point defects and complexes in the quaternary kesterite semiconductor Cu^2^ ZnSnS_4_. *Phys. Rev. B*. **81**, 245204 (2010).

[72] Varku, S., Pradhan, K. P. & Routray, S. Performance limitation of Cu2FeSnS4 solar cell: understanding impact of density of defect states. *Opt. Mater. (Amst)*. **133**, 112885 (2022).

[73] Wang, K. et al. Structural and elemental characterization of high efficiency Cu2ZnSnS4 solar cells. *Appl. Phys. Lett.* **98**, (2011).

[74] Sobayel, K. et al. Efficiency enhancement of CIGS solar cell by WS2 as window layer through numerical modelling tool. *Sol. Energy*. **207**, 479–485 (2020).

[75] Sravani, L., Routray, S. & Pradhan, K. P. Toward quantum efficiency enhancement of kesterite nanostructured absorber: a prospective of carrier quantization effect. *Appl. Phys. Lett.* **117**, (2020).

[76] Chandrasekar, P., Palaniswamy, S. K. & Routray, S. Exploiting high-density Earth-Abundant Kesterite Quantum Wells for Next-Generation PV technology. *IEEE Trans. Electron. Devices*. **68**, 5511–5517 (2021).

[77] Sahoo, G. S., Routray, S., Pradhan, K. P. & Mishra, G. P. Electrical, Optical, and reliability analysis of QD-Embedded Kesterite Solar Cell. *IEEE Trans. Electron. Devices*. **68**, 5518–5524 (2021).

[78] Izadi, F., Ghobadi, A., Gharaati, A., Minbashi, M. & Hajjiah, A. Effect of interface defects on high efficient perovskite solar cells. *Optik (Stuttg)*. **227**, 166061 (2021).

[79] Chouhan, A. S., Jasti, N. P. & Avasthi, S. Effect of interface defect density on performance of perovskite solar cell: correlation of simulation and experiment. *Mater. Lett.* **221**, 150–153 (2018).

[80] Kumar, A. & Thakur, A. D. Role of contact work function, back surface field, and conduction band offset in Cu _2_ ZnSnS _4_ solar cell. *Jpn J. Appl. Phys.* **57**, 08RC05 (2018).

[81] Dharmadasa, I. M., Bunning, J. D., Samantilleke, A. P. & Shen, T. Effects of multi-defects at metal/semiconductor interfaces on electrical properties and their influence on stability and lifetime of thin film solar cells. *Sol. Energy Mater. Sol. Cells*. **86**, 373–384 (2005).

[82] Haddout, A., Raidou, A. & Fahoume, M. Influence of the layer parameters on the performance of the CdTe solar cells. *Optoelectron. Lett.* **14**, 98–103 (2018).

[83] SINGH, P., SINGH, S., LAL, M. & HUSAIN, M. Temperature dependence of I–V characteristics and performance parameters of silicon solar cell. *Sol. Energy Mater. Sol. Cells*. **92**, 1611–1616 (2008).

[84] Al Zoubi, T., Al-Gharram, M. & Moustafa, M. Insights into the impact of defect states and temperature on the performance of kesterite-based thin-film solar cells. *Optik (Stuttg)*. **264**, 169442 (2022).

